# Initial Temporal Muscle Thickness and Area: Poor Predictors of Neurological Outcome in Aneurysmal Subarachnoid Hemorrhage in a Central European Patient Cohort

**DOI:** 10.3390/jcm12165210

**Published:** 2023-08-10

**Authors:** Cihat Karadag, Marcel A. Kamp, Igor Fischer, Hieronymus D. Boogaarts, Kerim Beseoglu, Sajjad Muhammad, Jan F. Cornelius, Björn B. Hofmann

**Affiliations:** 1 Department of Neurosurgery, Medical Faculty and University Hospital Düsseldorf, Heinrich-Heine-University Düsseldorf, Moorenstr. 5, 40225 Düsseldorf, Germany; 2 Centre for Palliative and Neuro-Palliative Care, Brandenburg Medical School Theodor Fontane, Campus Rüdersdorf, 15562 Rüdersdorf bei Berlin, Germany; 3 Department of Neurosurgery, Medical Faculty, Radboud University Nijmegen, Geert Grooteplein Zuid 10, 6525 GA Nijmegen, The Netherlands

**Keywords:** aneurysmal subarachnoid hemorrhage, outcome prediction, sarcopenia, physical fitness

## Abstract

The temporalis muscle area (TMA) has been proclaimed as a surrogate parameter for estimating skeletal muscle mass. Pilot studies in Asian populations suggested temporal muscle thickness (TMT) and TMA as prognostic factors for neurological outcomes in aneurysmal subarachnoid hemorrhage (aSAH) patients. This study aimed to validate these findings in a larger European patient cohort. We retrospectively analyzed age, sex, aneurysm location, treatment, World Federation of Neurosurgical Societies (WFNS) grade, Fisher score, and modified Rankin Score (mRS) at six months in patients with aSAH. TMT and TMA measurements were obtained from initial native CT scans. Logistic regression with the dichotomized six-month mRS as the outcome incorporating TMT, weighted average of TMT, or TMA as predictors was performed. Of the included 478 patients, 66% were female, the mean age was 56, and 48% of patients had an mRS of three to six after six months. The mean TMT at the level of the Sylvian fissure was 5.9 (±1.7) mm in males and 4.8 (±1.8) mm in females. The mean TMA was 234.5 (±107.9) mm^2^ in females and 380 (±134.1) mm^2^ in males. WFNS grade (*p* < 0.001), Fisher score (*p* < 0.001), and age (*p* < 0.05) correlated significantly with the mRS after six months. No correlation was found between mRS after six months and the TMT at the Sylvian fissure (*p* = 0.3), the weighted average of TMT (*p* = 0.1), or the TMA (*p* = 0.1). In this central European patient cohort of 478 individuals, no significant associations were found between TMT/TMA and neurological outcomes following aSAH. Further prospective studies in diverse patient populations are necessary to determine the prognostic value of TMA and TMT in aSAH patients.

## 1. Introduction

Aneurysmal subarachnoid hemorrhage (aSAH) is a devastating neurological condition caused by the rupture of a brain aneurysm that leads to bleeding into the subarachnoid space [1]. This type of hemorrhage can result in significant morbidity and mortality, with survivors often facing long-term neurological deficits [2,3,4,5]. Early identification of patients at risk of poor outcomes is crucial for optimizing their management and improving their prognosis [6]. 

Currently, established clinical outcome parameters that can be rapidly recorded, such as the World Federation of Neurosurgical Societies (WFNS) grade, or radiological outcome parameters, such as the Fisher Score, are commonly used in clinical practice for prognostication in aSAH patients [7,8]. More complex parameters, such as the Mean Transit Time (MTT) or the heterogeneity of the MTT of computed tomography perfusion imaging, are also gaining acceptance [9,10,11,12,13]. However, the use of complex prediction models that can predict outcomes or complications more accurately than individual parameters has not yet become routine due to their more complicated application [14,15]. With the increasing digitization of healthcare, these models are likely to become more prevalent in the future. To enhance their accuracy, it is essential to explore potential predictive parameters for patient outcomes. 

Recent studies have suggested that skeletal muscle mass may be a useful prognostic marker in critically ill patients. The loss of muscle mass, known as sarcopenia, is associated with poor outcomes in various medical and surgical conditions, including brain metastasis, head and neck carcinoma, chronic subdural hemorrhage, intracranial aneurysm, stroke, and glioblastoma [16,17,18,19,20,21]. However, clinical diagnosis of sarcopenia, including measuring walking speed or hand strength, can be challenging to implement in aSAH patients with neurological deficits or impaired vigilance [22]. 

Given the frequent performance of multiple cranial CT scans in aSAH patients, radiographic measurement of the temporalis muscle has recently been proposed as a feasible alternative for sarcopenia diagnosis, as they may serve as correlates of skeletal muscle mass [23]. Retrospective pilot studies by Katsuki and Lim et al. have suggested that Temporalis Muscle Thickness (TMT) and Temporalis Muscle Area (TMA) could serve as independent predictors of clinical outcomes after aSAH [24,25,26,27]. To confirm this hypothesis, further evaluation is necessary.

Thus, the aim of this study was to investigate the extent to which TMT and TMA could serve as radiologic outcome predictors after aSAH, using a large European patient cohort.

## 2. Materials and Methods

All human participant procedures conducted in this study were consistent with the ethical standards of the institutional committee and adhered to the 1964 Helsinki Declaration and its subsequent revisions. The study received approval from the local ethics committee of the Medical Faculty of the Heinrich-Heine University, Düsseldorf, Germany (study ID: 2022-2175), and the committee waived formal consent requirements due to the study design. The data will be made available upon reasonable request. We followed the Strengthening the Reporting of Observational Studies in Epidemiology (STROBE) guidelines and the Equator Network’s recommendation for developing scientific manuscripts when preparing the manuscript.

### 2.1. Inclusion and Exclusion Criteria

We conducted a monocentric, retrospective study that included all patients with aneurysmal subarachnoid hemorrhage admitted to the Neurosurgical Department of the University Hospital Düsseldorf between 1 January 2015 and 1 October 2022. The study enrolled patients who met the following inclusion criteria: Suspicion of aSAH in the initial native CT scan, further approved by the presence of an aneurysm on CT angiography or digital subtraction angiography. Patients were excluded if they: (1) Lacked a pretreatment native CT scan that could assess the thickness and surface area of the temporal muscle, (2) had incomplete or impaired visualization of the muscle, e.g., due to skull fractures, or (3) had missing 6-month follow-up data.

### 2.2. Measurement of the Temporal Muscle

**Figure 1:**
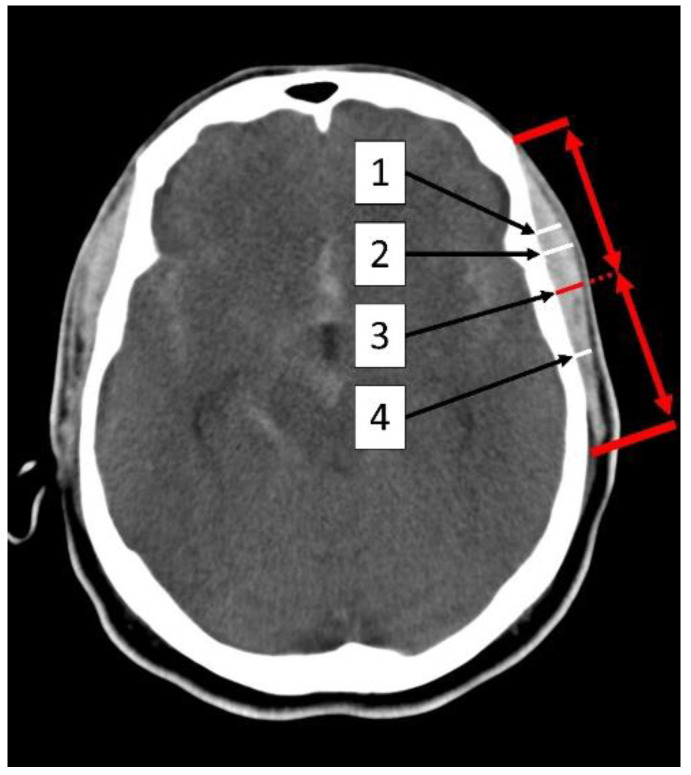
Illustration of TMT measurement in the initial native CT imaging (1) at the level of the Sylvian fissure, (2) at the thickest point, (3) at the mid-level of the muscle, and (4) at the inferior level of the Sylvian fissure. TMA and TMT were measured 5 mm above the orbital roof. The red arrows symbolize the mid-level of the temporal muscle.

The CT scans were performed using a multisectioned CT scanner (Somatom Volume Zoom, Definition Flash or AS; Siemens, Munich, Germany). The tilt of the axial native CT scan was standardized parallel to the skull base before being used to measure the dimensions of the temporal muscle. The TMT and the TMA were evaluated manually by a single rater using the Picture Archiving and Communication System (PACS) imaging software (Sectra IDS7, Version 24.1, Linkoping, Sweden), blinded to the patient’s outcomes. The temporal muscle was fully visualized bilaterally in a horizontal orientation in the native CT, 5 mm above the superior wall of the orbit. The TMA was evaluated manually by tracing the outline of the temporal muscle. The TMT was measured at four different locations in relation to the fronto-occipital orientation: (1) At the level of the Sylvian fissure, (2) at the thickest point, (3) at the mid-level of the temporal muscle, and (4) at the inferior level of the Sylvian fissure. These different TMT measurements are illustrated in Figure 1. TMT and TMA measurements were made on both the right and left temporal muscles for each patient. The average of the right and left temporal muscle means were used as the TMT and TMA for further statistical analysis.

### 2.3. Data Management and Definition of Outcome Measures 

Demographic data, including information on age at diagnosis, gender, location of the ruptured aneurysm, treatment, WFNS grade, Fisher score, modified Rankin Scale (mRS) at discharge, and mRS at 6 months, were collected retrospectively from patients’ charts. The mRS at 6 months after aSAH was used as the primary parameter for determining clinical outcomes. 

### 2.4. Statistical Analysis and Significance Level

With the exception of average age (reported as median and mean), all parameters were reported as the mean only. The standard deviation (SD) was reported for all descriptive statistics. The patient cohort was dichotomized for the analysis using the modified mRS and the TMT. Subgroup dichotomization by mRS at 6 months defined an mRS of 0–2 as favorable and an mRS of 3–6 as a poor clinical outcome. Subgroup dichotomization by TMT at the level of the Sylvian fissure was performed as low-volume TMT (TMT measurement smaller than the mean of all TMT measurements) and high-volume TMT (TMT measurement greater than the mean of all TMT measurements). We used the chi-squared test to test whether the distribution of other nominal variables differs between the two groups (Table 1). Furthermore, we used multiple logistic regression to model the dependence between predictor variables, including temporal muscle measurements and patient outcomes. Principal component analysis (PCA) was performed on temporal muscle measurements, and the projection on the first principal component was used as the predictor representing temporal muscle geometry. Age, sex, WFNS grade, and Fisher score were used as additional predictors. Patients whose data were not available for a certain parameter were excluded from the calculation of that parameter. A *p*-value less than 0.05 was considered significant.

## 3. Results

### 3.1. Patients

From 1 January 2015 to 1 October 2022, 707 patients were treated for aSAH at the Neurosurgical Department of Heinrich Heine University. Among these patients, pretreatment native CT scans were not available for eight patients, the complete visualization of the temporal muscle in the native axial CT scan was not possible for 74 patients, and the six-month mRS was not available for 147 patients. Therefore, 478 patients (66% female) with a mean age of 56 ± 13 were included in this study. Of these, 54% were treated surgically, and 34% received endovascular treatment. 55% of the patients presented with good grades (WFNS 1–3) and 45% with poor grades (WFNS 4–5) aSAH. A good clinical outcome at discharge (mRS 0–2) was found in 39%, while 61% had poor outcomes (mRS 3–6). After six months, 52% of the patients had a good outcome (mRS 0–2), and 48% had a poor outcome (mRS 3–6). Further patient characteristics are depicted in detail in Table 1, left column. 

### 3.2. Established Outcome Parameters

There was a significant correlation between patient age (*p* < 0.001), WFNS grade (*p* < 0.001), and Fisher score (*p* < 0.001) with the outcome measured by the dichotomized six-month mRS (Table 1). Younger patients and those with lower WFNS grades or Fisher scores had a higher probability of a good clinical outcome (mRS 0–2) six months after aSAH (Table 1).

### 3.3. Temporal Muscle Thickness

At the level of the Sylvian fissure, the mean temporal muscle thickness (TMT) was 5.2 ± 1.8 mm, while at the thickest point, it was 5.8 ± 1.8 mm. The mid-level of the temporal muscle had a mean TMT of 4.5 ± 1.6 mm, and the TMT at the inferior level of the Sylvian fissure was 2.7 ± 1.4 mm. No significant differences were found between the TMT measurements of the right and left sides (*p* = 0.93 for the TMT at the level of the Sylvian fissure). Male patients had a significantly larger TMT at the Sylvian fissure than female patients (*p* < 0.001). Age was significantly correlated with low and high-volume TMT at the Sylvian fissure (*p* < 0.001): The temporal muscle was thicker in younger patients (Table 2, left column).

**Figure 2:**
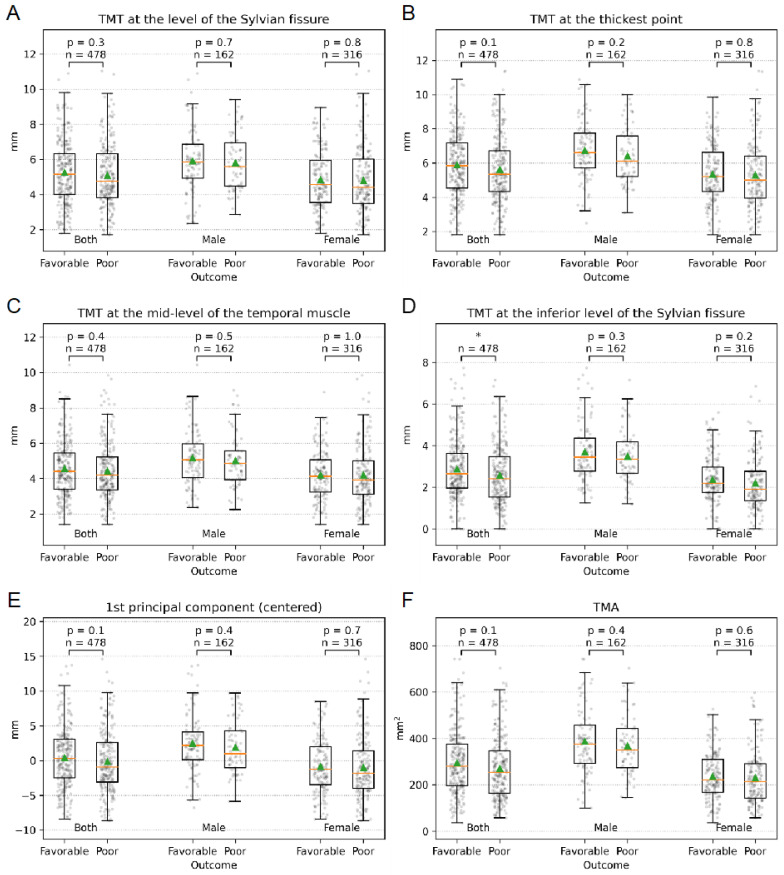
Temporalis muscle measurements in relation to patient outcomes. This figure presents the mean ± SD measurements of the temporalis muscle thickness (TMT) at various levels—the level of Sylvian fissure (**A**), the thickest point (**B**), the mid-level of the temporal muscle (**C**), and the inferior level of Sylvian fissure (**D**)—as well as the first principal component of TMT (**E**) and the temporal muscle area (TMA) (**F**). The measurements were taken in millimeters (mm). The results are displayed for the entire study population and patients with favorable vs. poor outcomes and are divided by sex. Significant differences (* *p* < 0.05) between the groups are indicated. Green triangle symbolizes the mean, the orange line the median. n, number of patients.

There were no significant associations between the TMT at the Sylvian fissure (*p* = 0.3), at the thickest point (*p* = 0.1), at the mid-level (*p* = 0.4) and the subgroups of patients with favorable (mRS 0–2) and poor (mRS 3–6) clinical outcomes (Figure 2A–C). A significant association was found between the TMT at the inferior level of Sylvian fissure and the subgroups of patients with favorable and poor outcomes (*p* = 0.02) (Figure 2D). However, there was no correlation between the projection on the first principal component (PC) of TMT and the dichotomized clinical outcome (*p* = 0.1) (Figure 2E).

After dichotomizing the patients into low and high-volume TMT at the Sylvian fissure, there was still no correlation between the subgroups and the mRS score after six months (*p* = 0.08). In addition, low and high-volume TMT at Sylvian fissure were not associated with aneurysm location (*p* = 0.7), WFNS score (*p* = 0.4), or Fisher grade (*p* = 0.8) (Table 2).

### 3.4. Temporal Muscle Area

The TMA was 283.8 mm^2^ (±136.1) (Table 2). Patients with favorable outcomes (mRS 0–2) had a TMA of 295.5 mm^2^ (±136.6), and the subgroup with poor outcomes (mRS 3–6) had a TMA of 271.3 mm^2^ (±134.8). No significant associations were found between TMA and the subgroups of patients with favorable (mRS 0–2) and poor (mRS 3–6) clinical outcomes (*p* = 0.1) (Figure 2F).

### 3.5. Multiple Logistic Regression Model

**Figure 3:**
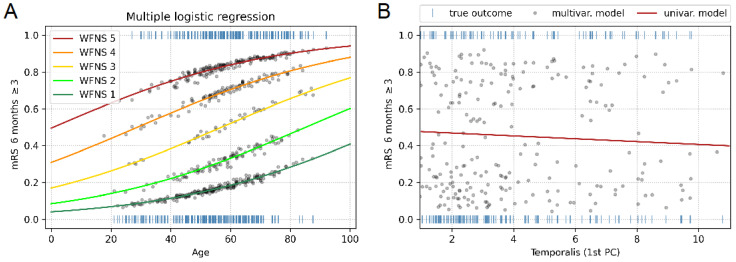
Multiple and univariate logistic regression analysis of dichotomized modified mRS scores at 6 months. (**A**) Blue vertical dashes represent observed outcomes, and colored lines show the probabilities modeled using age and the World Federation of Neurological Surgeons (WFNS) as predictors. Gray dots show the modeled probabilities using sex and the first principal component of temporalis muscle thickness (TMT) as additional predictors. The two models produce very similar results, indicating that neither TMT nor sex significantly affects the outcome. (**B**) Blue vertical dashes represent observed outcomes, and gray dots show the modeled probabilities using the multivariate model with age and WFNS as predictors. The red line shows the modeled probabilities using the univariate model with the first principal component of TMT as the predictor. mRS, modified Rankin scale; PC, Principal component.

Modeled probabilities using age, WFNS score, sex, and the first PC of TMT as predictors did not show improved results compared to probabilities modeled using only age and WFNS score as predictors. This suggests that neither TMT nor sex significantly affects the outcome. The relationship is shown graphically in Figure 3A. Figure 3B depicts the lack of predictiveness of the first PC of TMT in a univariate model (R2 = 0.005, *p* = 0.13) compared to the predictiveness of the multivariate model (R2 = 0.311, *p* = 0.001).

## 4. Discussion

Our study aimed to explore the predictive value of initial TMT and TMA in patients with aSAH for the neurologic outcome. Our large central European cohort included 478 patients with aSAH. Our findings demonstrated no significant association between TMT at the level of the Sylvian fissure, at mid-level, at the thickest point, or TMA and clinical outcome, as measured by dichotomized modified mRS after six months (Table 1, Figure 2A–C and Figure 3). A significant correlation between the TMT at the inferior level of the Sylvian fissure and clinical outcome was found (Table 1, Figure 2D). However, no significant association was found between the projection on the first principal component (PC) of TMT and the clinical outcome (*p* = 0.1) (Figure 2E). Consistent with previous studies, age, WFNS grade, and Fisher score were significant predictors of the six-month mRS in patients with aSAH (Table 1). 

In the perpetual pursuit of new and better prognostic assessments of aSAH patient outcomes, TMT and TMA have recently come into focus as possible correlates of sarcopenia. As previously described, the basis for this consideration is the observation of current studies in a multitude of diseases that skeletal muscle mass may be a prognostic factor in critically ill patients. Due to the potentially existing physical and mental impairment, the usual diagnostic criteria of sarcopenia cannot be used for patients with aSAH. Recently, the TMT and TMA have been shown to be possible surrogate parameters for skeletal muscle mass and sarcopenia risk [23,28,29,30], as at least one native CT of the skull is performed as part of diagnosing aSAH, which can be used for their determination. 

The value of TMT and TMA as a potential prognostic factor has already been examined in a multitude of diseases where, as in aSAH patients, there is cranial imaging at the beginning of therapy, and physical or mental impairments may exist. For instance, An et al. demonstrated in a study of 177 glioblastoma patients that thicker TMTs (>median) are associated with higher overall survival (OS) and progression-free survival (PFS) [31]. Broen et al. found sex-specific TMT cutoffs to be predictive of OS and PFS in 328 glioblastoma patients [32]. However, Wende et al. failed to find a significant correlation between TMT and OS in their study of 335 glioblastoma patients [33]. In patients with brain metastases, thicker TMTs (>median) were associated with higher OS in studies of melanoma (146 patients) [34], and a median-based sex-related TMT cutoff in non-small cell lung cancer (221 patients) was identified as a potential prognostic factor [16]. TMT has also been investigated as a predictor of clinical outcomes in other pathologies, including primary central nervous system lymphoma (128 patients), head and neck squamous cell carcinoma (106 patients), chronic subdural hematoma (171 patients), and acute ischemic stroke (265 patients) [17,20,21,35]. However, the results are often contradictory, as, for example, in stroke patients, variations of TMT in terms of OS or mRS after three months show different results in different patient cohorts [17,30]. In conclusion, while TMT and TMA are gaining increased attention, there remains a high degree of heterogeneity in the definition of measurement parameters, statistical analysis procedures, subgroup creation, and outcome parameters across studies. 

The morphology of the initial temporalis muscle in patients with aSAH presents a complex picture in the preliminary data collected and evaluated thus far. Lim et al. conducted a study of 51 aSAH patients to examine the association between TMT and clinical outcomes. They found that patients with TMT < 5.5 mm were more likely to experience DCI and overall complications and had a worse clinical outcome than patients with TMT ≥ 5.5 [27]. However, no significant correlation was found between TMT and mRS after 12 months. Similarly, Katsuki et al. studied 127 aSAH patients and found significant associations between a good clinical outcome and WFNS score, age, TMT, TMA, and smoking habits [24]. A significant correlation between the temporalis muscle and the mRS was found in 49 elderly aSAH patients (>75 years) as well as in 298 aSAH patients who received endovascular coiling [25,26]. Rodrigues et al. found a significant correlation between TMT/TMA and mRS at discharge/after 6 months in a study including 361 patients with intracranial aneurysms (199 ruptured, 162 unruptured) [19]. In contrast to these findings, our patient cohort showed no significant prognostic benefit for either TMT or TMA. Notably, we measured TMT at four standardized and validated different locations, whereas the aforementioned studies only measured TMT at the level of the Sylvian fissure. Our analysis found no significant correlation between TMT at the Sylvian fissure, at mid-level, at the thickest point, and mRS at six months (Figure 2 and Figure 3, Table 1). 

The differences between the observations in this study and the previous data from Katsuki et al. and Rodrigues et al. may be attributed to multiple underlying factors. Our data represents the largest patient cohort to date, with a focus on the European patient population. It is worth noting that the previous studies were based on significantly smaller patient cohorts, ranging from 49 to 298 patients. Additionally, there were significant differences in patients’ ethnicity, age, and type of therapy used across the studies. Notably, there was heterogeneity in the definition of measurement parameters, statistical analysis procedures, subgroup creation, and outcome parameters across the studies, making comparability and replication of findings difficult. For example, the definition of high vs. low volume TMT subgroups varied by sex and whether the median TMT of the study population or 2.5 SD below the mean TMT of the normative population was used. These inconsistencies and the very specific and different subgroup analyses in each study may hinder the comparability and replication of findings, highlighting the need for standardization in future research. 

Our study findings are consistent with current data from 293 western-European patients with aSAH, which suggest that skeletal muscle atrophy and myosteatosis have no significant impact on outcome (mRS at six months) [36]. It should be emphasized that this study did not use TMT or TMA to assess initial muscle mass. Instead, cross-sectional muscle measurements were taken at the level of the third cervical vertebra. Therefore, in two larger European patient cohorts, measured using different radiological surrogate parameters, the initial muscular mass did not have prognostic value for clinical outcome after six months in affected patients.

An important aspect to consider is the timing of CT imaging, which is crucial for interpreting TMT/TMA measurements, given the multifaceted pathophysiological underpinnings of sarcopenia. If sarcopenia is detected on the initial CT scan upon patient admission, it is likely to be associated with pre-existing conditions that could influence the subsequent outcomes, including frailty, diminished immune response, impaired physical functionality, and compromised nutritional status. This initial measurement thus provides a snapshot of the patient’s pre-admission health state, offering valuable information about their potential resilience or vulnerability to the unfolding disease process. In the examined patient cohort with aSAH, we do not observe a statistically significant correlation between this pre-admission health state and the neurological outcome.

On the other hand, the development of sarcopenia after ICU admission typically indicates a catabolic state directly linked to the severity of the illness. This catabolic condition may arise due to the impact of neurological injury, systemic inflammatory response, infectious complications, and specific pathologies such as critical illness myopathy. The resulting muscle loss may reflect the significant physiological stress endured during aSAH or the intensive care period, which can have a substantial impact on the patient’s recovery trajectory. Exactly this correlation of a decreasing temporal muscle volume over the course of intensive care therapy in relation to the severity of aSAH has recently been demonstrated by Kofler et al. in their study [37]. 

The timing of TMT/TMA measurements during the course of aSAH is, therefore, crucial for the interpretation and prognostic implications of the measurements, as the presence or development of sarcopenia may potentially provide clinicians with insights into the patient’s health status and possible challenges they face during their treatment and recovery.

While our study provides valuable insights, there are some limitations that should be considered when interpreting the results. Firstly, this study was conducted in a single center, using a retrospective design, which may have introduced information and selection biases, and only focused on the initial TMT and TMA, despite possible changes over the time of treatment. Further multicentric and prospective studies are needed to fully evaluate the temporal muscle as an independent outcome predictor. Furthermore, as this study is retrospective in nature, certain parameters, such as nutrition management and specialized laboratory-chemical values like Albumin, could not be measured or included. It is unclear whether TMT and TMA are affected by other factors, such as parotitis, and it was not possible to rule out idiopathic muscle atrophy or hypertrophy in individual patients. Additionally, despite not reaching statistical significance, our analysis revealed consistent numerical disparities in TMT/TMA variables between groups. Specifically, patients with a favorable neurological outcome (mRS 0–2) exhibited numerically higher TMT/TMA values, albeit large standard deviations. The lack of statistical significance might, therefore, hint at a lack of power in the study despite the large patient cohort, suggesting the necessity for an even larger patient sample size. Finally, the clinical outcome was assessed using the mRS after six months, and long-term follow-up data were not included in this study. Future studies with longer follow-up periods may be needed to fully evaluate the prognostic value of skeletal muscle measurements in patients with aSAH.

## 5. Conclusions

In our study with 478 aneurysmal SAH patients, no correlation between TMT or TMA and clinical outcome after six months measured by the dichotomized mRS was found. Temporal muscle morphology, therefore, does not appear to be a suitable prognostic factor for outcome in European aneurysmal SAH patients. Further prospective and multicentric studies with standardized assessment approaches are necessary to provide a more complete understanding of the role of temporal muscular mass in aneurysmal SAH outcomes.

## Figures and Tables

**Table 1:** Patient characteristics and the difference between favorable and poor outcomes.

Baseline Characteristics	Total n = 478	mRS 0–2 n = 247	mRS 3–6 n = 231	*p* -Value
**Sex**				
**Male**	162 (34%)	95 (38%)	67 (29%)	
**Female**	316 (66%)	152 (62%)	164 (71%)	
**Age (years)**				
**mean ± SD**	56 ± 13	53 ± 13	60 ± 13	**<0.001**
**<20**	7 (1%)	6 (2%)	1 (0%)	
**<30**	29 (6%)	20 (8%)	9 (4%)	
**<40**	50 (10%)	32 (13%)	18 (8%)	
**<50**	129 (27%)	80 (32%)	49 (21%)	
**<60**	145 (30%)	73 (30%)	72 (31%)	
**<70**	77 (16%)	31 (13%)	46 (20%)	
**<80**	35 (7%)	4 (2%)	31 (13%)	
**<90**	6 (1%)	1 (0%)	5 (2%)	
**Aneurysm location**				
**Acom**	175(37%)	99 (40%)	76 (33%)	
**ACA**	11 (2%)	7 (3%)	4 (2%)	
**PCA**	17 (4%)	9 (4%)	8 (3%)	
**MCA**	107 (22%)	56 (23%)	51 (22%)	
**ICA**	32 (7%)	14 (6%)	18 (8%)	
**Pcom**	50 (10%)	31 (13%)	19 (8%)	
**BA**	38 (8%)	12 (5%)	26 (11%)	
**VA**	10 (2%)	3 (1%)	7 (3%)	
**PICA**	18 (4%)	7 (3%)	11 (5%)	
**SCA**	1 (0.2%)	1 (0.4%)	0 (0)	
**Multiple locations**	19	8	11	
**WFNS scale**				
**mean ± SD**	2.9 ± 1.7	2 ± 1.4	3.9 ± 1.4	**<0.001**
**1–3**	261 (55%)	196 (79%)	65 (28%)	
**4–5**	217 (45%)	51 (21%)	166 (72%)	
**Fisher scale**				
**mean ± SD**	3.1 ± 0.9	2.8 ± 1	3.5 ± 0.6	**<0.001**
**0–2**	64 (13%)	59 (24%)	5 (2%)	
**3–4**	414 (87%)	188 (76%)	226 (98%)	
**Treatment**				
**1 Surgical**	259 (54%)	144 (58%)	115 (50%)	
**2 Endovascular**	164 (34%)	96 (39%)	68 (29%)	
**3 None**	55 (12%)	7 (3%)	48 (21%)	
**mRS at discharge**				
**mean ± SD**	3.5 ± 2.1	1.8 ± 1.3	5.3 ± 1	**<0.001**
**0–2**	187 (39%)	186 (76%)	1 (0%)	
**3–6**	290 (61%)	60 (24%)	230 (100%)	
**No information**	1	1	0	
**mRS 6 months**				
**mean ± SD**	2.9 ± 2.4	0.8 ± 0.7	5.1 ± 1.2	**<0.001**
**0–2**	247 (52%)	247 (100%)	0 (0%)	
**3–6**	231 (48%)	0 (0%)	231 (100%)	
**TMA**				
**Total**	283.8 ± 136.1	295.5 ± 136.6	271.3 ± 134.8	0.1
**Female**	234.5 ± 107.9	237.8 ± 99.7	231.4 ± 115.3	0.6
**Male**	380.0 ± 134.1	387.9 ± 137.2	368.9 ± 129.8	0.4
**TMT Sylvian fissure**				
**Total**	5.2 ± 1.8	5.3 ±1.8	5.1 ± 1.8	0.3
**Female**	4.8 ±1.8	4.8 ± 1.7	4.8 ± 1.8	0.8
**Male**	5.9 ± 1.7	5.9 ±1.8	5.8 ±1.6	0.7
**TMT at the thickest point**				
**Total**	5.8 ± 1.8	5.9 ± 1.8	5.6 ± 1.9	0.1
**Female**	5.3 ± 1.8	5.4 ± 1.7	5.3 ± 1.9	0.8
**Male**	6.6 ± 1.7	6.7 ± 1.7	6.4 ± 1.6	0.2
**TMT at the mid-level of the muscle**				
**Total**	4.5 ± 1.6	4.6 ± 1.5	4.4 ± 1.6	0.4
**Female**	4.2 ± 1.5	4.2 ± 1.4	4.2 ± 1.6	1
**Male**	5.1 ± 1.6	5.2 ± 1.6	5.0 ± 1.5	0.5
**TMT at the inferior level of the Sylvian fissure**				
**Total**	2.7 ±1.4	2.9 ± 1.4	2.6 ± 1.4	0.02
**Female**	2.3 ± 1.2	2.4 ± 1.1	2.2 ± 1.3	0.2
**Male**	3.6 ± 1.4	3.7 ± 1.4	3.5 ± 1.3	0.3

Subgroup dichotomization by mRS at 6 months defined an mRS of 0–2 as favorable and an mRS of 3–6 as a poor clinical outcome. M. temporalis measurements depicted in mean (± Standard Deviation); Acom: Anterior communicating artery, ACA: Anterior cerebral artery, AICA: Anterior inferior cerebellar artery, BA: Basilar artery, ICA: Internal carotid artery, MCA: Middle cerebral artery, mRS: Modified Rankin score, PCA: Posterior cerebral artery, Pcom: Posterior communicating artery, PICA: Posterior inferior cerebellar artery, SCA: Superior cerebellar artery, SD: Standard deviation, TMA: Temporal muscle area, TMT: Temporal muscle thickness, VA: Vertebral artery, WFNS scale: World Federation of Neurosurgical Societies scale.

**Table 2:** Comparison between high volume and low volume TMT.

Baseline Characteristics	Total n = 478	Low-Volume < Median n = 244	High-Volume ≥ Median n = 234	*p* -Value
**Sex**				
**Male**	162 (34%)	53 (22%)	109 (47%)	
**Female**	316 (66%)	191 (78%)	125 (53%)	
**Age (years)**				
**mean ± SD**	56 ± 13	59 ± 12	53 ± 14	**<0.001**
**<20**	7 (1%)	2 (1%)	5 (2%)	
**<30**	29 (6%)	8 (3%)	21 (9%)	
**<40**	50 (10%)	13 (5%)	37 (17%)	
**<50**	129 (27%)	68 (28%)	61 (27%)	
**<60**	145 (30%)	80 (33%)	65 (26%)	
**<70**	77 (16%)	46 (19%)	31 (13%)	
**<80**	35 (7%)	25 (10%)	11 (5%)	
**<90**	6 (1%)	2 (1%)	3 (1%)	
**WFNS scale**				
**mean ± SD**	2.9 ± 1.7	3 ± 1.7	2.8 ± 1.7	0.42
**1–3**	261 (55%)	128 (52%)	133 (57%)	
**4–5**	217 (45%)	116 (48%)	101 (43%)	
**Fisher scale**				
**mean ± SD**	3.1 ± 0.9	3.2 ± 0.8	3.1 ± 0.9	0.8
**0–2**	64 (13%)	32 (13%)	32 (14%)	
**3–4**	414 (87%)	212 (87%)	202 (86%)	
**Treatment**				
**1 Surgical**	259 (54%)	124 (51%)	135 (58%)	
**2 Endovascular**	164 (34%)	82 (34%)	82 (35%)	
**3 None**	55 (12%)	38 (16%)	17 (7%)	
**mRS at discharge**				
**mean ± SD**	3.5 ± 2.1	3.7 ± 2.1	3.2 ± 2.1	0.1
**0–2**	187 (39%)	86 (35%)	101 (43%)	
**3–6**	290 (61%)	157 (65%)	133 (57%)	
**No information**	1	1	0	
**mRS 6 months**				
**mean ± SD**	2.9 ± 2.4	3.2 ± 2.4	2.7 ± 2.3	0.08
**0–2**	247 (52%)	116 (48%)	131 (56%)	
**3–6**	231 (48%)	128 (52%)	103 (44%)	

Sex, age, aneurysm location, WFNS scale, Fisher score, mRS at discharge, mRS after six months of patients with low- vs. high-volume TMT at the Sylvian fissure. mRS: Modified Rankin score, SD: Standard deviation, WFNS scale: World Federation of Neurosurgical Societies scale.

## Data Availability

The datasets used and/or analyzed during the current study are available from the corresponding author upon reasonable request.
